# Antifungal Activity of Brazilian Propolis Microparticles against Yeasts Isolated from Vulvovaginal Candidiasis

**DOI:** 10.1093/ecam/neq029

**Published:** 2011-03-09

**Authors:** Kelen Fátima Dalben Dota, Marcia Edilaine Lopes Consolaro, Terezinha Inez Estivalet Svidzinski, Marcos Luciano Bruschi

**Affiliations:** ^1^Graduate Program of Health Sciences, State University of Maringa, Parana, Brazil; ^2^Department of Pharmacy, State University of Maringa, Colombo Avenue, 5790, CEP 87020-900, Maringa, Parana, Brazil

## Abstract

Propolis, a resinous compound produced by *Apis mellifera* L. bees, is known to possess a variety of biological activities and is applied in the therapy of various infectious diseases. The aim of this study was to evaluate the *in vitro* antifungal activity of propolis ethanol extract (PE) and propolis microparticles (PMs) obtained from a sample of Brazilian propolis against clinical yeast isolates of importance in the vulvovaginal candidiasis (VVC). PE was used to prepare the microparticles. Yeast isolates (*n* = 89), obtained from vaginal exudates of patients with VVC, were exposed to the PE and the PMs. Moreover, the main antifungal drugs used in the treatment of VVC (Fluconazole, Voriconazole, Itraconazole, Ketoconazole, Miconazole and Amphotericin B) were also tested. Minimum inhibitory concentration (MIC) was determined according to the standard broth microdilution method. Some *Candida albicans* isolates showed resistance or dose-dependent susceptibility for the azolic drugs and Amphotericin B. Non-*C. albicans* isolates showed more resistance and dose-dependent susceptibility for the azolic drugs than *C. albicans*. However, all of them were sensitive or dose-dependent susceptible for Amphotericin B. All yeasts were inhibited by PE and PMs, with small variation, independent of the species of yeast. The overall results provided important information for the potential application of PMs in the therapy of VVC and the possible prevention of the occurrence of new symptomatic episodes.

## 1. Introduction

Vulvovaginal candidiasis (VVC) is a disease caused by abnormal growth of yeast-like fungi in the mucosa of the female genital tract, classified by the World Health Organization as a sexually transmitted disease of frequent sexual transmission [1]. VVC is caused mainly by the genus Candida, the major agent being *Candida albicans*, and the prevalence of this yeast can reach 85–95% [2]. Moreover, studies have shown the increasing infections by non-*C. albicans* species (*C. tropicalis, C. glabrata, C. krusei, C. parapsilosis, C. pseudotropicalis, C. lusitaniae*) in VVC [3, 4]. Because the disease strikes millions of women annually, leading to great discomfort, interfering with sexual and affective relations and impairing work performance, it has been considered an important worldwide public health concern [2]. VVC is the first cause of vulvovaginitis in Europe and the second in the USA and Brazil. It represents 20–25% of the vaginal discharges of infectious nature. It is estimated that about 75% of the adult women show at least one episode of VVC during their lifetimes, 40–50% of those will experience new surges and 5% will reach the recurrent character (RVVC), defined as the occurrence of four or more symptomatic episodes in a one year interval [4].

In recent years, drug-resistance to antifungal agents and optimizing therapy of Candida infections have been broadly focused [5]. Moreover, the therapeutic arsenal available for the treatment of fungal infections is quite restricted, being limited to polyenic and azolic antifungal chemicals [6, 7]. For the treatment of VVC, nystatin (cream or vaginal ovule) has been used for almost three decades, but therapeutic fails were observed [8, 9]. Amphotericin B would be an excellent therapeutic resource because of its high efficacy, but has showed high toxicity [9]. The azolics are drugs that inhibit the synthesis of ergosterol, an important component of the fungal cell membrane [7]. Of these, Fluconazol (FLU) is one of the most used in VVC, but, in addition to its high cost, it has been reported the development of resistance of *C. albicans* and non-*C. albicans* yeasts against this drug [2]. These facts make the management of patients with VVC and RVVC difficult and put into evidence the need of searching for new, effective, safe, low-cost antifungal alternatives against this pathology.

Natural products have been traditionally used in the treatment of diseases because they are source of many active compounds. Propolis, a strongly adhesive resinous compound produced by *Apis mellifera* L. bees, has demonstrated important antimicrobial activity and has been used to treat inflammatory, bacterial and fungal diseases [10–14]. With complex chemical composition, typically consisting of waxes, resins, water, inorganics, phenolics and essential oils, the exact composition of propolis is dependent upon the source plant(s) [10, 15–20]. Thus, Brazilian propolis has been the subject of intensive research over the last few decades [21, 22]. It has been sub-divided into four types based on the association of ethanol extracts of Brazilian samples with the levels of bioactive compounds [23]. Brazilian propolis is known for its antifungal activities [12, 13, 24].

Furthermore, propolis ethanol extract (PE), alone or incorporated in another dosage form, is commonly utilized as therapeutics [10, 11, 25]. However, the high ethanol concentration is a disadvantage of PE, resulting in difficulties on the administration and incorporation in some dosage forms like vaginal ointments [11, 25]. The patient compliance to the therapeutics is committed too. Studies have shown that it is possible to obtain microparticles containing propolis without the high ethanol concentration and with prolonged release of propolis [11, 12, 26].

Despite this discovery and the problem of VVC, no studies have been carried out to determine the inhibitory effect of Brazilian propolis microparticles (PMs) against vaginal pathogens. Thus, the aim of this study was to prepare and evaluate the *in vitro* antifungal activity of PMs against yeasts (*C. albicans* and non-*C. albicans*) of VVC importance.

## 2. Methods

### 2.1. Chemicals and Reagents

Type A gelatin, Royal (São Paulo, Brazil), was used without further purification. FLU and Voriconazole (VORI) were obtained from Pfizer Inc (New York, NY, USA). Itraconazole (ITRA), Ketoconazole (KETO) and Miconazole (MICO) were purchased from Janssen Pharmaceutical (Titusville, NJ, USA) and Amphotericin B (AMB) was obtained from Squibb Pharmaceutical (Princeton, NJ, USA). Furthermore, Sabouraud dextrose broth (SDB), Sabouraud dextrose agar (SDA) and Mueller Hinton broth (MHB) were purchased from Difco (Detroit, USA). CHROMagar Candida was purchased from Probac (Paris, France) and RPMI-1640 medium from Sigma (Steinheim, Germany). All other chemicals and solvents were of analytical grade and purchased from Merck (Darmstadt, Germany).

### 2.2. Propolis Sample

The Brazilian propolis sample was collected from hives of *Apis mellifera* L. bees at the Iguatemi Experimental Farm, which belongs to the State University of Maringá (FEI-UEM), Maringá, Paraná state, Brazil. The apiary was located north-west of Paraná State, inside a eucalyptus reserve surrounded by native forest with a predominance of *Baccharis dracunculifolia* (Asteraceae). Propolis collection was carried out both inside and outside the hive, with the surfaces being scraped smoothly with a wooden chisel. The samples were combined into a single batch, packed in a sealed plastic bag and stored at –20°C.

### 2.3. Preparation and Characterization of PE

Propolis was powdered and the PE was prepared with propolis/ethanol ratio of 30/70 (w/w) by turbo extraction [11]. Exactly weighted 10 g of PE was concentrated on water bath (100°C) with eventual shaking. The concentrated material was dried on the Ohaus-MB 200 infrared analytical balance (Pine Brook, NJ, USA) at 110°C and the result was presented as “dryness residue” (DR) value. Six replicates were carried out to estimate the inherent variability of each the determination.

The total phenol content (TPC) of PE was determined by the Folin-Ciocalteau method [20]. PE was mixed with 6 mL of the Folin-Ciocalteau and 6 mL of 20% Na_2_CO_3_. After 2 h, the absorbance was measured by Shimadzu UV-1650PC spectrophotometer (Tokyo, Japan) at wavelength of 760 nm. A calibration curve with solutions of gallic acid was used as reference. TPC was expressed as percentage of total phenolic substances in PE and corresponds to mean of six replicates.

### 2.4. Preparation and Characterization of PMs

PE was dispersed in a gelatin solution at 20°C through the dripping technique using a syringe of 10 mL, and with magnetic agitation by 30 min [11]. The amount of gelatin utilized was a function of the PE DR. The ratio gelatin/PE DR was 6/1 (w/w). The final dispersion was spray-dried in a BÜCHI Mini Spray Dryer model B-191 (Büchi, Flawil, Switzerland) through the nozzle, using the following conditions: inlet temperature of 160°C; feed rate of 6%; aspiration of 80%; pressure of 3%; mannitol 20% (w/w). The resultant dried product was collected and stored in a vacuum desiccator at room temperature.

The mean particle size and size distribution of PMs were assessed by a CARL ZEISS optical microscope and the CARL ZEISS AxioVision Image Analysis System (CARL ZEISS, Germany). Particles were placed on glass slide and the size measurements of microparticles were performed using Feret's diameter as parameter. A total of 2000 PMs were measured and the particle size distribution was estimated. Moreover, the shape and surface of produced microparticles were investigated by scanning electron microscopy (SEM). The spray-dried products were fixed on supports and coated with gold-palladium under argon atmosphere using a gold sputter module in a high-vacuum evaporator. Samples were then observed with SHIMADZU SS550 scanning electron microscope (SHIMADZU, Tokyo, Japan) at 15 kV.

The amount of propolis incorporated into microparticles was determined by the Folin-Ciocalteau method [20], with minor modifications. PMs were mixed with 6 ml of the Folin-Ciocalteau and 6 mL of 20% Na_2_CO_3_. After 2 h, the absorbance was measured by Shimadzu UV-1650PC spectrophotometer (Tokyo, Japan) at wavelength of 760 nm. A calibration curve with solutions of gallic acid was used as reference. TPC was expressed as percentage of total phenolic substances in PMs and corresponds to mean of six replicates. The propolis encapsulation efficiency was calculated by comparing the TPC of the PE with that of the PMs.

### 2.5. Antifungal Assays

A total of 89 yeast strains, obtained from vaginal exudates of the VVC patients, were tested: 58 *C. albicans* and 31 non-*C. albicans* (17 *C. glabrata*, 01 *C. tropicalis*, 08 *C. guilliermondii* and 05 *C. parapsilosis*). These yeast cells were isolated and identified in 2007 [27, 28] and are part of a yeast bank from the Laboratory of Medical Mycology at the State University of Maringá (Maringá, PR, Brazil). The isolates were stored in SDB with 10% of glycerol at –20°C after identification.

The yeast isolates were tested by Clinical Laboratory Standards Institute reference broth, microdilution method for FLU and ITRA [29], with modifications for others drugs and for PE and PMs [30]. Stock solutions of drugs were prepared at 10 times the strength of final concentration and diluted with RPMI 1640, with l-glutamine, without bicarbonate, supplemented with 2% dextrose and buffered to pH 7.0 with 0.165 N-morpholinopropanesulfonic acid to obtain twice the final concentration. Yeast isolates were grown on SDA for 48 h, at 37°C. The density of suspension of cells in sterile distilled water was adjusted by spectrophotometer to a final transmission of 90%, at a wavelength of 530 nm. Suspension was made with a 1 : 50 dilution in sterile distilled water, followed by a 1 : 5 dilution in RPMI medium to obtain two times the final concentration. The test was performed in sterile, flat-bottom 96-well microtiter plates. Volumes of 100 *μ*L of twice the dilutions and 100 *μ*L of twice the inoculum were dispensed into wells. Inoculum size averaged between 0.5 and 2.5 × 10^3^ cells mL^–1^. For the PE and PMs test, 100 *μ*L aliquots of MHB were distributed from column 2 to 11 microtiter plates.

PMs were diluted (1.0 g of PMs, 1.0 mL of ethanol and 4.0 mL of sterile distilled water). PE or PMs, in 100 *μ*L aliquots, were added to columns 1 and 2 of the microplate and from column 2 onwards the serial dilution was performed at a ratio of 2 until the 10th well (dilutions of 1 : 1024). In this way, the concentrations of the tested PE ranged from 17.19 to 1100.63 *μ*g mL^–1^ of TPC, and from 10.86 to 5570.49 *μ*g mL^–1^ of TPC in PMs.

For each isolate, negative (only RPMI or MHB) and positive controls (RPMI/MHB and inoculate, without antifungal addition) of growth and the possible action of the diluent (alcohol and inoculate) were included. In each plate a strain of *C. parapsilosis* (ATCC 22019) was included as reference yeast. The plates thus mounted were incubated at 35°C for 48–72 h with daily monitoring. After 48 h the reading of the drug test was performed in microplate reader (Asys Hitech GmbH, Eugendorf, Austria), and after 72 h the reading of the PE and PMs was made through visual comparison, by mirror reflex.

The minimum inhibitory concentrations (MICs) of azoles were defined as the first well with a significant growth reduction (*∼*50%) when compared to that of positive control. In the case of AMB, it was defined as the lowest concentration capable of inhibiting 90% of the growth. Endpoints for antifungal agents: isolates with MIC between 16 and 32 *μ*g mL^–1^ for FLU, 0.25 and 0.5 *μ*g mL^–1^ for ITRA, KETO and MICO and 2 *μ*g mL^–1^ for VORI had reduced dose-dependent susceptibility. Isolates with MICs ≤8 *μ*g mL^–1^ for FLU, ≤0.125 *μ*g mL^–1^ for ITRA, KETO and MICO, and ≤1 *μ*g mL^–1^ for AMB and VORI, were susceptible. MICs ≥ 64 *μ*g mL^–1^ for FLU, ≥1 *μ*g mL^–1^ for ITRA, KETO and MICO, ≥4 *μ*g mL^–1^ for VORI and ≥2 *μ*g mL^–1^ for AMB, were resistant [6, 8].

For PE and PMs, the results of the MIC were considered relative to TPC and were determined as the lowest concentration of total phenols capable of inhibiting 100% of the yeast growth, as compared to its respective positive control [13].

## 3. Results

### 3.1. Characterization of PE

Dryness residue of PE was 18.43 ± 0.17% with 0.92% of relative standard deviation (RSD). TPC was 7.28 ± 0.12% and its RSD was 1.70%.

### 3.2. Characterization of Microparticles

**Figure 1:**
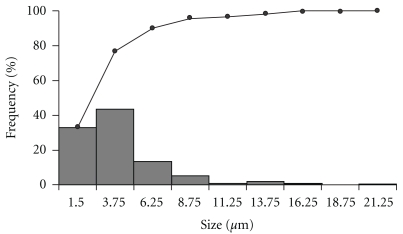
Size distribution of propolis microparticles (PMs): size frequency distribution (bars) and size cumulative frequency distribution (line). The particle size class interval is 2.5 *μ*m.

**Figure 2:**
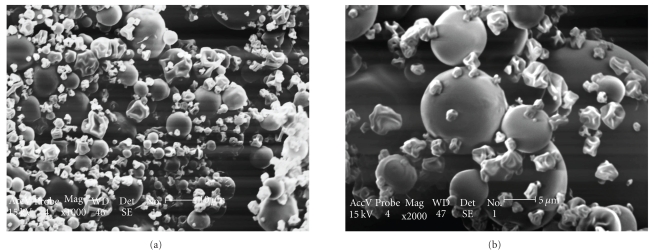
SEM images of spray-dried propolis microparticles showing the outer topology: (a) original magnification ×1000; (b) original magnification ×2000.

PMs were easily obtained by spray-drying technique [11]. Measurements of microparticles showed a narrow size distribution (Figure 1) and the mean particle size of 4.06 *μ*m.  Figure 2 indicated that microparticles were in fine spherical shape with smooth surfaces, with a low number of coalesced microparticles and low agglomeration determined by SEM. The amount of total phenolics compounds in PMs was 0.97 ± 0.04% and the propolis encapsulation efficiency into microparticles was 78.51 ± 2.81% (*n* = 6).

### 3.3. Antifungal Assay

Table 1 shows the interpretation of the MIC results for antifungal drugs as for sensitivity, dose-dependent susceptibility and resistance. Some isolates of *C. albicans* showed resistance for ITRA, KETO, MICO, and AMB (10.3, 25.8, 3.5 and 1.7%, resp.) and dose-dependent susceptibility for all azolics. Isolates of non-*C. albicans* showed more resistance and dose-dependent susceptibility than *C. albicans*. Tested isolates of non-*C. albicans* showed resistance for ITRA, KETO and MICO (29.0, 48.4, and 16.1%), but all were sensitive or dose-dependent susceptibility for AMB. More specifically, two isolates from *C. parapsilosis*, one *C. guilliermondii* and six *C. glabrata* were resistant for ITRA. For KETO, nine *C. glabrata*, five *C. guilliermondii* and one *C. parapsilosis* were resistant, and for MICO, three *C. glabrata* and two *C. parapsilosis* were resistant.

**Figure 3:**
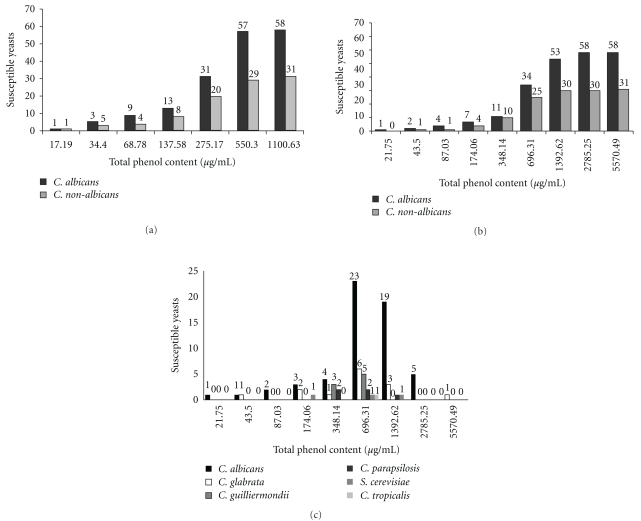
Cumulative numbers of the susceptibility of 89 yeasts by concentration of total phenolic compounds (*μ*g/mL), expressed for each observed minimum inhibitory concentration (MIC), to: (a) propolis extractive solution; (b) propolis microparticles; (c) propolis microparticles for yeast species.

Figure 3(a) shows that all the yeasts, both *C. albicans* and non-*C. albicans*, were inhibited by PE up to a maximal concentration of 1100.63 *μ*g mL^–1^ of TPC (average of 275.17 *μ*g mL^–1^). Most of the isolates (96.63%, *n* = 86) were inhibited by PE with concentration of TPC of 550.30 *μ*g mL^–1^.  Figure 3(b) shows that all the tested yeasts, both *C. albicans* and non-*C. albicans*, were also inhibited by PMs up to a maximal concentration of 5570.49 *μ*g mL^–1^ of TPC (average = 696.31 *μ*g mL^–1^). Most of the isolates (93.26%, *n* = 83) were inhibited by the PMs with concentration of TPC of 1392.62 *μ*g mL^–1^. Just one of the non-*C. albicans*, a *C. glabrata* isolate, required the TPC of 5570.49 *μ*g mL^–1^ in PMs (Figure 3(c)). The positive control of solvent (alcohol 96°GL) did not demonstrate any activity against the yeasts.

All resistant isolates of *C. albicans* were inhibited by PE with concentration of TPC from 137.58 to 550.30 *μ*g mL^–1^, and by PMs from 174.06 to 1392.62 *μ*g mL^–1^ (Figures 3(a) and 3(b)). Moreover, all resistant isolates of non-*C. albicans* were inhibited by PE between 34.40 and 1100.63 *μ*g mL^–1^ and by the PMs between 174.06 and 5570.49 *μ*g mL^–1^ (Figures 3(a), 3(b), and 3(c)).

## 4. Discussion

Propolis is a hive product containing chiefly beeswax and plant-derived substances such as resin and volatile compounds. The sample of propolis used in this study was collected from hives located inside a eucalyptus reserve surrounded by native forest with a predominance of *B. dracunculifolia*. This propolis is classified as “type BRP”, a typical propolis from north-west of Paraná State, Brazil [23]. Predominant components of the resin of this type of propolis are cinnamic acids, chiefly compounds bearing prenyl groups. Terpenoid compounds, such as sesqui, di and pentacyclic triterpenoids, have been detected in many, but not all, samples investigated. Brazilian propolis is a rich source of phenolic substances and the most of them are prenylated phenylpropanoids [11, 12, 19, 31, 32]. Phenolic compounds are identified as being responsible for anti-inflammatory, antimicrobial and in particular antifungal actions of propolis [13, 33, 34].

Gelatin microparticles containing propolis were prepared by spray-drying technique. PE characteristics, composition of dispersion and spray-drying conditions gave PMs with good surface and shape characteristics. Size analysis showed that obtained structures are microparticles. These PMs characteristics are useful for development of propolis vaginal dosage form without the high ethanol concentration of PE [11, 12, 26].

The Folin-Ciocalteau method was used to determine the TPC in the PE and PMs. Thus, we have used this method as a criterion just for judging the relative amount of propolis incorporated into the gelatin particles. Moreover, the obtained propolis encapsulation efficiency into microparticles can be attributed to the nature of the spray-drying mechanism, which provides high drug loading, particularly in the case of drying of solutions or well-stabilized suspensions [11, 35].

The combination of temperature, small space and humidity provide the beehives with good conditions for microbial growth [12]. Nevertheless, this does not occur because of the antimicrobial activity of propolis [13, 24]. The antimicrobial activity is the most popular of the propolis, being between its biological actions more extensively investigated [16, 36]. The Brazilian propolis produced where the main botanic source is *B. dracunculifolia* is highly recommended by modern herbalists since it displays microbicidal, anti-inflammatory, immunomodulatory and anti-ulcer properties [37]. Thus, antifungal activity of PE and PMs was evaluated by microdilution method against several yeasts isolated from patients with VVC, namely *C. albicans* and non-*C. albicans.* Studies of this kind are relevant because of the need of new therapeutic alternatives, especially low-cost, efficient and safe ones, for the treatment of VVC, considering the few therapeutic options and the resistance observed for some drugs [38].

Imhof et al. [14] tested PE at concentration of 5% in women with chronic vaginitis without laboratory definition of the causal agent, and concluded that this compound can be an alternative in these instances. However, the authors pointed to the need of studies comparing the effects of PE with classical antimicrobial therapies. In this sense, in the present study, the *in vitro* activity of various antifungal drugs was used as a parameter of comparison with PE and PMs activity, also *in vitro* (Table 1).

**Figure 4:**
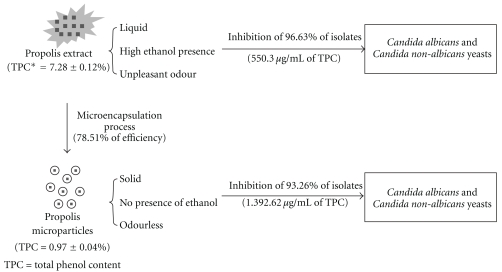
The hypothetical diagram for comparing the antimicrobial activity of propolis extract and propolis microparticles.

In relationship to the activity of the antifungal drugs against the tested yeasts, several isolates showed resistance and dose-dependent susceptibility for azolics, and some resistance for AMB. The strains of non-*C. albicans* showed more resistance and dose-dependent susceptibility than *C. albicans*. However, all the yeasts, both *C. albicans* and non-*C. albicans*, were inhibited by PE and PMs, showing that both are effective. For PMs, a bigger TPC was necessary than the PE, which did not alter the inhibition profile (Figure 3). Similar results were shown when propolis extractive solutions and their respective PMs were tested against oral pathogens [12]. Considering the PE dryness residue, the PE amount added to prepare the microparticles and the drug trapping efficiency of PMs, that was not greater than 78.51 ± 2.81%, the PMs activity was similar to the PE. The hypothetical diagram is presented in Figure 4. Moreover, the results of Oliveira et al. [13] showed a high sensitivity to PE against *C. albicans* and non-*C. albicans* isolated from patients with onychomycoses, evidencing an excellent efficacy.

It could be observed that the PE and PMs have shown very good results, with small variation, independent of the species of yeast isolated from VVC. This fact puts the PMs in a position comparable to classical antifungal compounds [39]. Even the resistant isolates of *C. albicans* and non-*C. albicans* were inhibited by the PMs, suggesting an action better than that of antifungal drugs tested. The presence of these resistant isolates and the high index of yeasts with dose-dependent susceptibility, especially non-*C. albicans* (Figure 3(b)), evidence a decreasing of the therapeutic expectation toward these antifungals. Studies of Consolaro et al. [8] and Ferraza et al. [3] also found an even higher incidence of yeasts with dose-dependent susceptibility (45.0 and 51.5%, resp.), but absence of resistance.

Several studies have reported that from 25 to 50% of the women bearing RVVC show re-incidence 4–6 weeks after conventional antifungal therapy with azolic compounds, making the management of this condition quite complicated [40, 41].

In conclusion, considering the antifungal activity showed by PMs and that the high ethanol concentration is a disadvantage of PE, this report clearly showed that PMs arises as a possible agent for the treatment and especially the prevention of the new symptomatic episode the VVC. Moreover PMs have the advantage of to be incorporated in some dosage forms, like vaginal ointments, and to be administered into the vaginal mucosa more easily and safely.

## Funding

Brazilian agency CAPES (*Coordenadoria de Aperfeiçoamento de Pessoal de Nível Superior*).

## Figures and Tables

**Table 1:** Interpretation of the results of MIC for antifungal drugs against vaginal yeasts of *C. albicans* (*n* = 58) and non-*C. albicans* (*n* = 31).

	*Candida albicans*						Non-*Candida albicans*					
Antifungals	S^a^		DDS^b^		R^c^		S		DDS		R	
	*n*	%	*n*	%	*n*	%	*n*	%	*n*	%	*n*	%
FLU	55	94.8	3	5.2	—	—	26	83.9	5	16.1	—	—
ITRA	48	82.8	4	6.9	6	10.3	18	58.1	4	12.9	9	29.0
KETO	20	34.5	23	9.7	15	25.8	9	29.0	7	22.6	15	48.4
MICO	42	72.4	14	24.1	2	3.5	6	19.4	20	64.5	5	16.1
VORI	57	98.3	1	1.7	—	—	30	96.8	1	3.2	—	—
AMB	57	98.3	—	—	1	1.7	31	100.0	—	—	—	—

DDS, dose-dependent susceptibility; R, resistant.

^a^S: Isolates with MIC ≤ 8  *μ*g mL^–1^ for FLU, ≤0.125 *μ*g mL^-1^ for ITRA, KETO, and MICO, and ≤1 *μ*g mL^–1^ for AMB and VORI, ^b^DDS: Endpoints for antifungal agents: isolates with MIC between 16 and 32 *μ*g mL^–1^ for FLU, 0.25–0.5 *μ*g mL^–1^ for ITRA, KETO, and MICO, and 2 *μ*g mL^–1^ for VORI, ^c^R: Isolates with MIC ≥ 64 *μ*g mL^–1^ for FLU, ≥1 *μ*g mL^–1^ for ITRA, KETO, and MICO, ≥ 4 *μ*g mL^–1^ for VORI and ≥2 *μ*g mL^–1^ for AMB.
